# High‐Performance Violet Phosphorene‐Based Device via Tellurium Doping‐Induced Contact Resistance Reduction

**DOI:** 10.1002/advs.77560

**Published:** 2026-09-27

**Authors:** Zhuorui Wen, Xuewen Zhao, Wenju Zhou, Wenqi Mu, Rui Zhai, Jiazheng Lin, Xin Liu, Kerui Hu, Zhiqi Zhang, Guodong Meng, Yonghong Cheng, Huiyang Gou, Jinying Zhang

**Affiliations:** ^1^ State Key Laboratory of Electrical Insulation and Power Equipment Center of Nanomaterials for Renewable Energy School of Electrical Engineering Xi'an Jiaotong University Xi'an Shanxi People's Republic of China; ^2^ State Grid Integrated Energy Service Group Co., Ltd. Beijing China; ^3^ Center for High Pressure Science and Technology Advanced Research (HPSTAR) Beijing China

**Keywords:** charge mobility, contact resistance, n‐type, p‐type, violet phosphorus

## Abstract

Two‐dimensional materials have attracted much attention in various research fields. The phosphorene structures with various allotropes, another type of elemental 2D materials after graphene, have been demonstrated to have unique optoelectronic and photoelectronic properties. However, an inert environment is required to measure phosphorene‐based field effect transistors. The experimentally obtained charge mobility from devices is significantly lower than the intrinsic values due to high contact resistance between phosphorene and electrodes. Here, tellurium atoms have been introduced for the first time to significantly lower the contact resistance, enabling measurements under ambient conditions to obtain high charge mobility. The crystal structure of violet tellurium phosphorus was determined by single‐crystal X‐ray diffraction to keep the same crystal structure as violet phosphorus, where the P9 position is occupied by tellurium/phosphorus atoms as a mixed Te1/P9 site. The p‐type violet phosphorene nanosheet (2.04 cm^2^ V^−1^ s^−1^) was switched into a high‐performance n‐type violet tellurium phosphorene nanosheet (134.12 cm^2^ V^−1^ s^−1^). The contact resistance between channel materials and electrodes was found to be significantly reduced from 550–860 MΩ for violet phosphorene nanosheets to 1.13–1.80 MΩ for violet tellurium phosphorene nanosheets due to tellurium substitution, resulting in robust performance of violet phosphorene under ambient conditions.

## Graphical abstract

Tellurium substitution for phosphorus in violet phosphorus significantly reduces contact resistance, enhancing FET performance under ambient conditions.

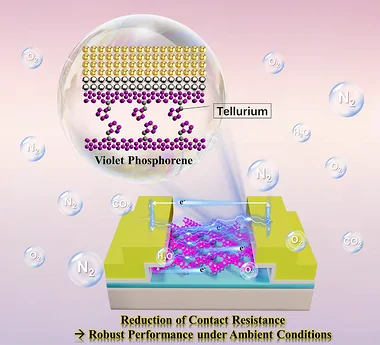

## 1 Introduction

Two‐dimensional (2D) materials exhibit unique properties in the fields of mechanics [1, 2], electronics [3, 4], and optics [5, 6, 7], and have great potential for various applications. Phosphorene structures, another type of elemental 2D material after graphene, have many allotropes such as black [8, 9], violet [10], and blue ones [11]. Phosphorene was found to be sensitive to air due to its lone electron pairs, where an inert environment is required for the operation of phosphorene‐based field effect transistors (FET)[12]. Violet phosphorus (VP), the most stable phosphorus allotrope [10], was demonstrated to overcome various limitations of graphene, transition metal dichalcogenides, and black phosphorus. Violet phosphorene has been shown to be a promising 2D material for electronic [13] and optoelectronic devices [14, 15, 16, 17]. The violet phosphorene was predicted to have high anisotropic hole mobility of 3000−7000 cm^2^ V^−1^ s^−1^ [18]. The charge mobility of violet phosphorene has been measured by time‐resolved absorption spectroscopy to be as high as 2100 cm^2^ V^−1^ s^−1^ along the a‐axis [19]. However, the charge mobility of violet phosphorene nanosheets (VPNS) measured from FET were found to be significantly lower than theoretical ones. The phosphorene‐based FET were also found to be easily damaged during measurements under ambient conditions [13, 20, 21]. High contact resistance between phosphorene and electrodes is one of the critical factors for the extremely low performance of FET [22, 23].

Doping has been demonstrated to be able to modulate carrier type and reduce the contact resistance between channel materials and electrodes, thereby significantly enhancing the performance of FET devices. The tellurium doping was found to obtain higher mobility and better stability of black phosphorus‐based FET under ambient conditions [24]. The Vanadium doping was found to gradually switch the p‐type of molybdenum disulfide into n‐type with increasing doping and enhance its charge carrier mobility [25]. Arsenic doping was also introduced into black phosphorus to tune its band gap from 0.3 to 0.15 eV, thereby improving its electronic and optical properties [26]. The selenium doping was also found to enhance the contact between black phosphorus and metal electrodes to modulate its Schottky barrier width, enhancing the optoelectronic performance of black phosphorus [27].

The precise determination of the crystal structure of channel materials either with or without doping is crucial for a deep understanding and optimization of their performance. However, the growth of high‐quality single crystals to determine their crystal structures by single‐crystal X‐ray diffraction (SC‐XRD) is always a big challenge. The crystal structure of black phosphorus was successfully determined by SC‐XRD in 1965 [28]. However, the accurate doping of black phosphorus (such as arsenic or antimony) hasn't been determined by SC‐XRD due to the difficulty of producing high‐quality single crystals. The crystal structures of VP [10] and antimony‐substituted violet phosphorus (violet antimony phosphorus) [29, 30] were determined by SC‐XRD from macro‐sized high‐quality single crystals.

Herein, tellurium has been introduced to substitute some phosphorus of violet phosphorus to significantly reduce the contact resistance between the channel materials and electrodes, enhancing the performance of FET even under ambient conditions. The tellurium‐substituted VP (violet tellurium phosphorus, VPTe) single crystals have been successfully produced. The crystal structure of VPTe was determined by SC‐XRD (P_80_Te, CSD‐2449898), where the vertex position of [P9] is occupied by a mixed site of Te/P. The p‐type VP was switched into n‐type VPTe, where the measured charge mobility was found to be significantly enhanced.

## 2 Results and Discussion

**FIGURE 1:**
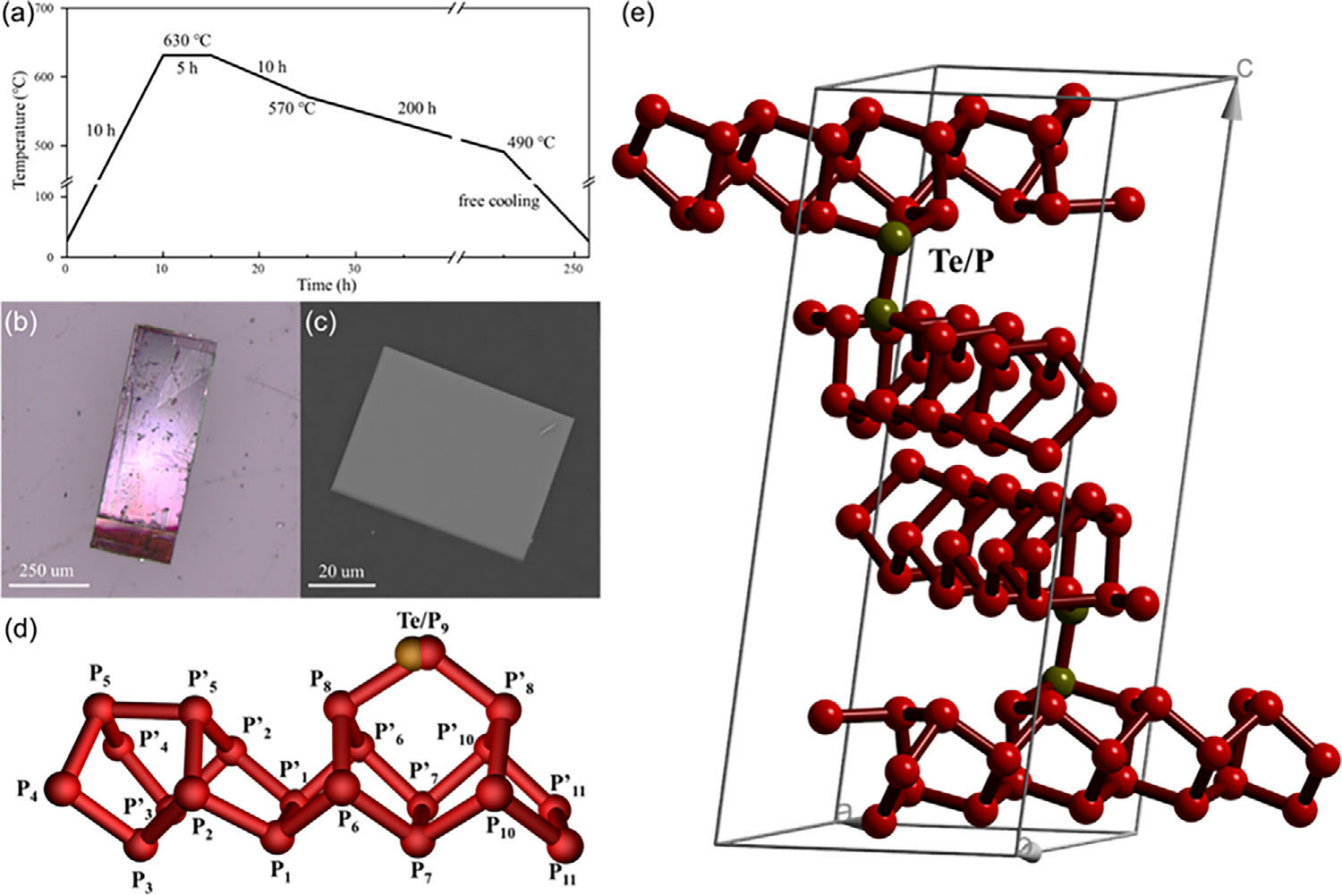
(a) Synthesis parameters for VPTe crystals. (b) Optical and (c) SEM images of a VPTe single crystal. (d) Atomic positions in the P_80_Te single crystal structure. (e) Crystal structure of P_80_Te single crystals.

The violet tellurium phosphorus (VPTe) single crystals were prepared by a modified chemical vapor transport (CVT) method [31]. Briefly, a mixture of tin powder, amorphous red phosphorus, and tellurium tetraiodide powder was sealed in a vacuum quartz tube and then heated in a muffle furnace according to synthesis parameters shown in Figure S1. The yield was optimized with a reagent mixture of 200 mg tin powder, 470 mg amorphous red phosphorus, and 600 mg tellurium tetraiodide powder with optimized synthesis parameters to obtain high‐quality single crystals with macro‐sizes (Figure S2). The sample was first heated to 630 °C for 10 h, held for 5 h, and then cooled to 570 °C with a cooling rate of 0.1 °C/min. The sample was then cooled from 570 °C to 490 °C for about 200 h, and then furnace cooled to room temperature (Figure 1a). The reacted quartz tube was then removed from the muffle furnace, where multi‐crystals with a metal grey color were observed (Figure S3). The VPTe crystals with clear dark red luster (Figure S4) were picked out after cracking the metal grey crystals. The VPTe single crystal was observed to have a regular rectangular shape and flat, well‐defined facets with a lateral size of about 1 mm (Figure 1b). The layered structure of VPTe crystals was easily observed from scanning electron microscopy (SEM) (Figure 1c). The crystal structure of the VPTe crystals was determined by SC‐XRD to be monoclinic P_80_Te with a space group of P2/n (CSD‐2449898, a = 9.1903 Å, b = 9.1174 Å, c = 21.8094 Å, β = 97.745^°^). The detailed SC‐XRD information of P_80_Te is shown in Table S1. One phosphorus atom position (Figure 1d, P9) is now occupied by a tellurium/phosphorus (Te/P) atom, forming a mixed Te1/P9 occupied site (Figure 1e). The specific coordinates of each phosphorus and tellurium atom in the P_80_Te single crystal are shown in Table S2. Some of the phosphorus atoms of VP were successfully replaced by tellurium to give VPTe with the same crystal structure as VP [10], whose lattice constants were only slightly varied.

**FIGURE 2:**
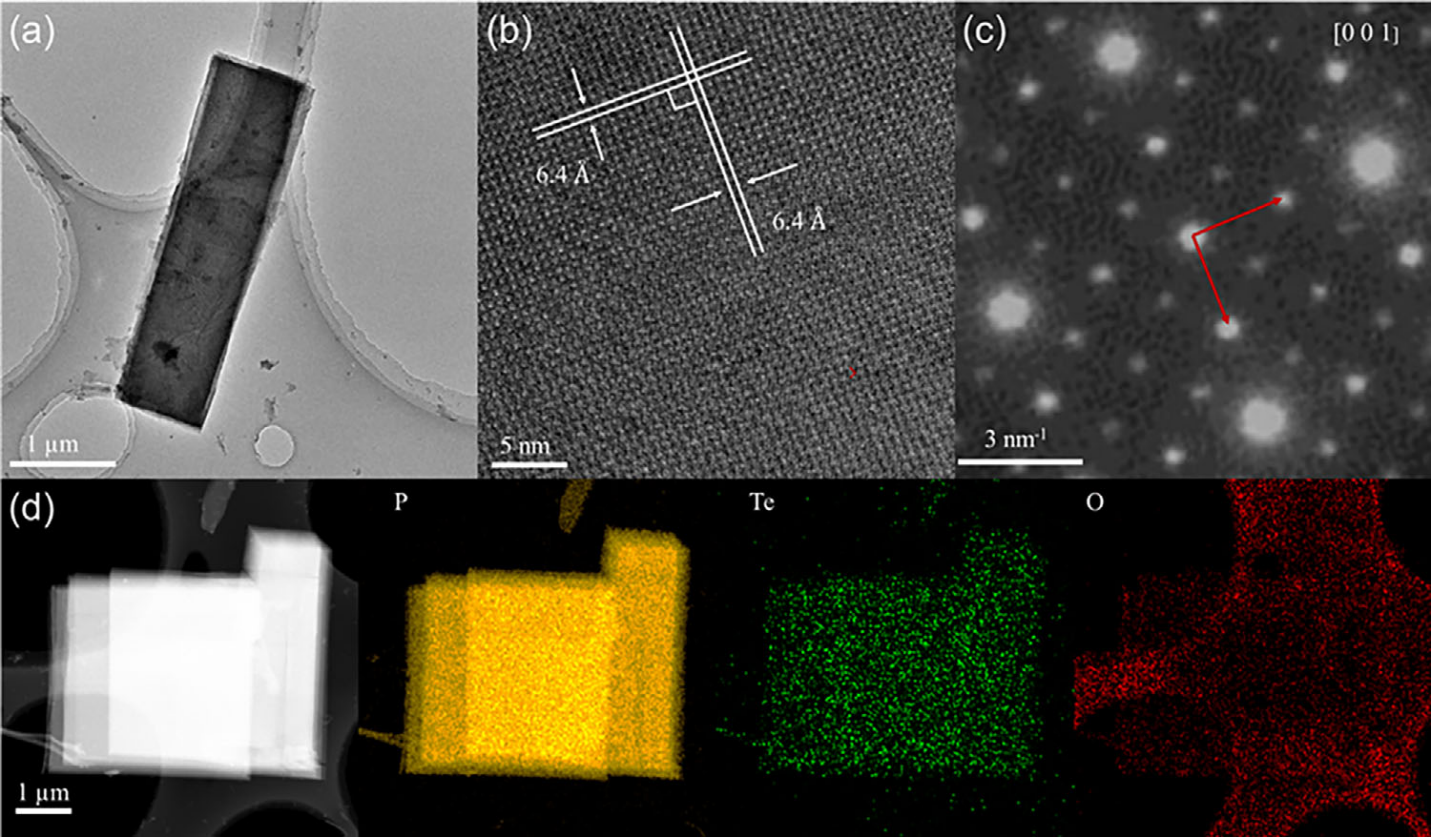
(a) HRTEM images of exfoliated VPTeNS and (b) corresponding enlarged structures. (c) SAED pattern with [001] as the zone axis. (d) HAADF image of VPTeNS and corresponding elemental mapping analysis.

Similar to VPNS [32], the exfoliated VPTeNS were observed to exhibit rectangular shapes with perpendicular edges by transmission electron microscopy (TEM) (Figure 2a). Two sets of perpendicular crystal planes with interplanar spacing of 6.4 Å corresponding to the (110) and (1$\bar{1}$0) lattice planes of P_80_Te were observed from a high‐resolution transmission electron microscope (HRTEM) image (Figure 2b). The interplanar spacing and interplanar angle obtained from the selected area electron diffraction (SAED) (Figure 2c) are also well consistent with the diffraction pattern of the P_80_Te lattice determined from SC‐XRD with a zone axis of [001]. The VPTeNS was also demonstrated to have a homogeneous distribution of phosphorus and tellurium elements by high‐angle annular dark field (HAADF) imaging and elemental mapping analysis (Figure 2d); almost no oxidation was detected from VPTeNS even after solvent exfoliation under ambient conditions.

**FIGURE 3:**
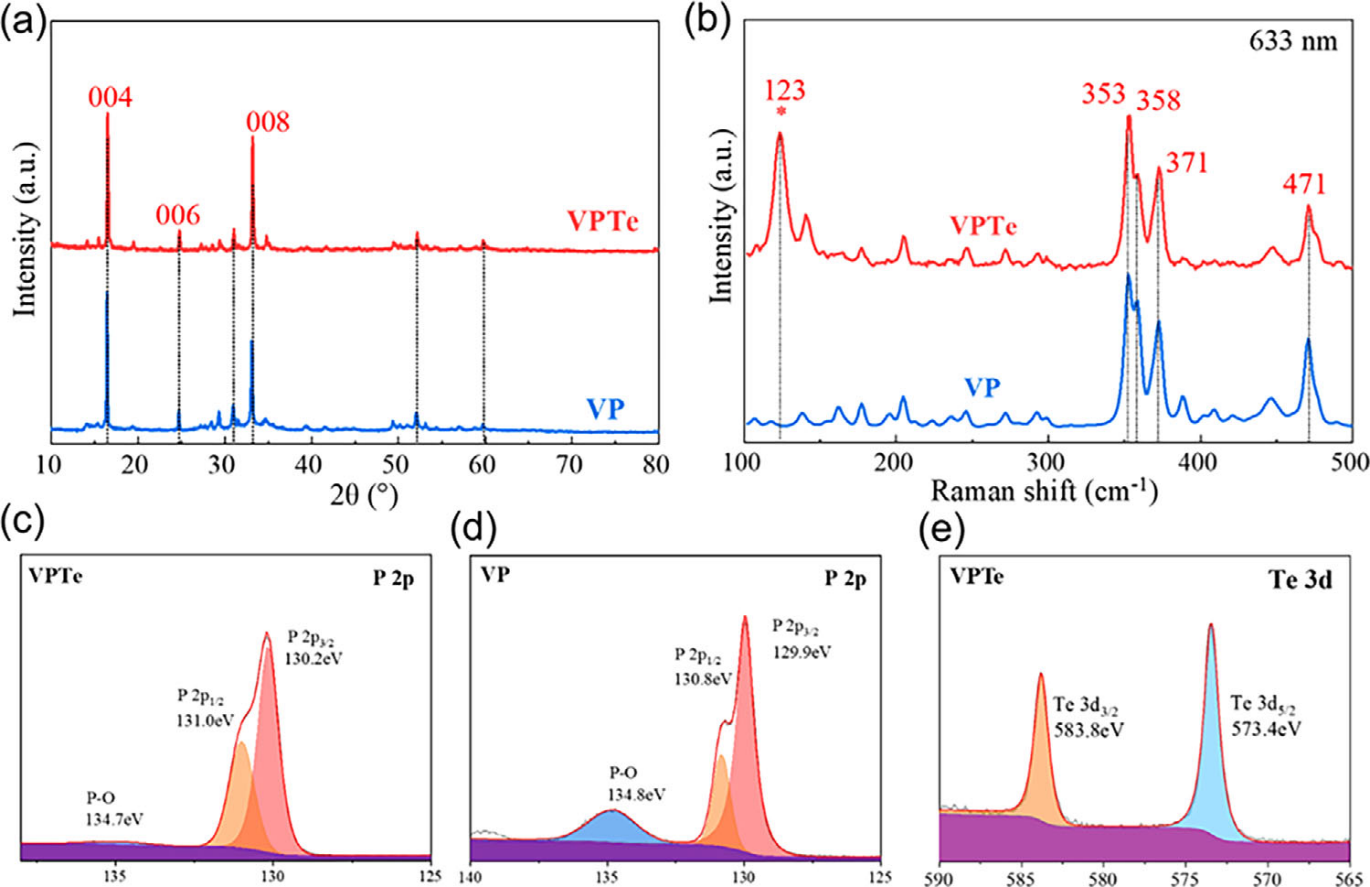
(a) XRD patterns and (b) Raman spectra of VPTe (red) and VP (blue). High‐resolution P2p of (c) VPTe and (d) VP. (e) High‐resolution Te3d of VPTe.

The structure of VPTe was further analyzed using powder X‐ray diffraction (XRD), Raman scattering, and high‐resolution X‐ray photoelectron spectroscopy (XPS). Strong XRD peaks with 2θ at 16.5^°^, 24.8^°^, and 33.2^°^, corresponding to the {004}, {006}, and {008} were detected from VPTe powder (Figure 3a, red). The reflection peaks of VPTe were found to be slightly shifted to a higher angle compared to those of VP (Figure 3a, black), indicating slightly smaller inter‐layer spacing was obtained due to the introduction of Te substitution. Raman characterization of VPTe and VP single crystals using a 633 nm excitation laser is shown in Figure 3b. Stretching vibrational modes of the [P9] cage at 353 cm^−1^ (S^2^_[P9]_), the stretching modes of [P8] at 358 cm^−1^ (S^1^_[P8]_) and 371 cm^−1^ (S^2^_[P8]_) at the two characteristic peaks of the stretching mode, and [P9] tangential stretching mode along the tube axis (Tg) at 471 cm^−1^ were detected [10, 33]. An additional weak Raman peak at 123 cm^−1^ was observed from the VPTe single crystal (Figure 3b, red), which might be due to localized defect modes induced by tellurium doping or triggered splitting of P‐P vibrational modes [31, 34]. The Raman characterization of VPTe single crystals further confirms the successful substitution of tellurium in VPTe single crystals. Two strong P 2p_3/2_ and P 2p_1/2_ peaks with binding energies of 131.0 and 130.2 eV corresponding to elemental phosphorus and an extremely weak peak with a binding energy of 134.7 eV corresponding to the P‐O were detected from VPTe crystals, indicating that the oxidation of VPTe is negligible. The binding energies of P2p_3/2_ and P2p_1/2_ in VPTe were found to be 0.2 eV higher than those of VP crystals (Figure 3d) due to the partial substitution of phosphorus atoms by tellurium atoms, where electrons were slightly transferred from phosphorus to Te. The additional Te 3d binding energies of VPTe were observed to be located at 583.8 eV (Te3d_3/2_) and 573.4 eV (Te3d_5/2_) (Figure 3e), slightly smaller than elemental Te (Te3d_3/2_: Te3d_5/2_) [35]. The slight shift of Te 3d is well consistent with its electron doping results, further validating the successful substitution of tellurium atoms in VP.

**FIGURE 4:**
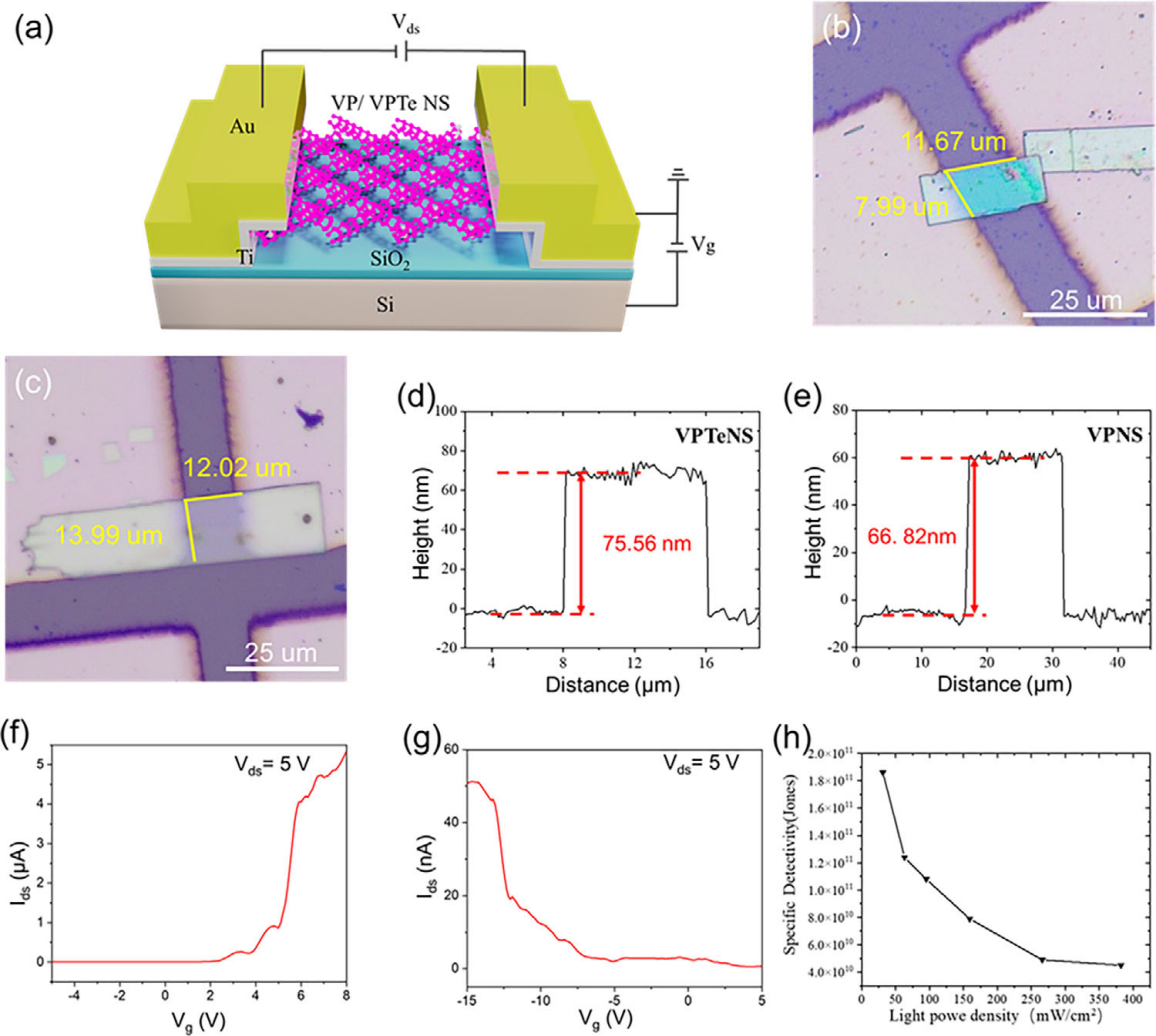
(a) Schematic diagram of a VPNS/VPTeNS‐based FET. Optical image of a (b) VPTeNS‐based and (c) VPNS‐based FET. Height profile of the corresponding (d) VPTeNS and (e) VPNS. Transfer characteristic curves of (f) VPTeNS‐based and (g) VPNS‐based FET. (h) Specific detectivity of VPTeNS with different incident light at V_ds_ = 1 V.

FET based on mechanically exfoliated VPTeNS and VPNS were fabricated to investigate their electronic properties. A bottom‐gated FET was fabricated using exfoliated VPTeNS or VPNS as channel materials. The nanosheets were mechanically transferred onto Si substrates with a 300 nm thick thermally oxidized silica layer via a blue tape‐assisted mechanical exfoliation technique [13]. The source and drain contacts were patterned using a copper gate mask technique [36, 37], followed by the deposition of 10 nm thick titanium and 50 nm thick gold layers. The FET devices were then annealed at 300 °C for 120 s under a nitrogen atmosphere. The device structure is schematically shown in Figure 4a. The optical image of the FET prepared based on VPTeNS is shown in Figure 4b, featuring a conducting channel with a length of 11.67 µm and a width of 7.99 µm. The channel length of the VPNS was measured to be 12.02 µm with a width of 13.99 µm (Figure 4c). The thickness of the VPTeNS and VPNS was measured by atomic force microscopy (AFM) to be 75.56 nm (Figure 4d) and 66.82 nm (Figure 4e), respectively.

The transfer characteristics of FETs based on VPTeNS and VPNS were measured under ambient conditions. The VPTeNS was detected to be an n‐type semiconductor (Figure 4f), while the VPNS was demonstrated to be characteristic p‐type (Figure 4g), which is consistent with reported ones [13, 21]. The p‐type VP has been demonstrated to be switched into n‐type VPTe by tellurium substitution. The charge mobility of the VPTeNS and VPNS‐based FETs was calculated according to Equation S1 [38]. A high electron mobility of 134.12 cm^2^ V^−1^ s^−1^ (thickness: 75.56 nm) was extracted from the transfer curves of the VPTeNS‐based FET, significantly higher than that of the VPNS FET (2.04 cm^2^ V^−1^ s^−1^, thickness: 66.82 nm). The charge mobility of VPTe was found to be comparable to that of VPAs (137.06 cm^2^ V^−1^ s^−1^) [39] with a higher on/off ratio (896 vs 664) and a smaller subthreshold swing (109 mV/dec vs 114 mV/dec) (Table S3). The charge mobility of VPTe was found to be higher than that of VPSb (58.96 cm^2^ V^−1^ s^−1^) [29] and much higher than that of pristine ones [13, 39].

An optical band gap of 1.22 eV was measured from VPTe by UV/Vis diffuse reflectance spectroscopy, slightly smaller than that of VP (Figure S7a). The conduction‐band minimum (CBM) and valence‐band maximum (VBM) were further deduced from its VB‐XPS measurements (Figure S7b) [7, 40]. The CBM was found to shift from −3.71 eV of VP to −4.26 eV of VPTe due to Te substitution, while the VBM shifted from −5.34 eV of VP to −5.48 eV of VPTe (Figure S7c). The reduced bandgap and the downward CBM shift facilitate carrier excitation and injection, decreasing the effective transport barrier and contact resistance. The carriers are then more efficiently accumulated and transported through the VPTe channel, yielding a significantly enhanced field‐effect mobility compared with pristine VP.

The air stability of P_80_Te phosphorene nanosheet‐based FET was found to be much higher than reported phosphorene‐based ones. The black phosphorene‐based FETs were required to be conducted under an inert atmosphere. The P_80_Te phosphorene nanosheet‐based FET were found to be able to be performed under ambient conditions. The performance variation of P_80_Te phosphorene nanosheet‐based FET were monitored according to air exposure time with V_ds_ = 5 V to evaluate its air stability (Figure S8a‐c). The corresponding carrier mobilities were found to decrease from 119.52 cm^2^ V^−1^ s^−1^ before air exposure to 108.03 cm^2^ V^−1^ s^−1^ after 12 h of air exposure and 90.86 cm^2^ V^−1^ s^−1^ after 24 h of air exposure. The VPTe FET was found to still maintain a relatively high carrier mobility and clear transfer characteristics after 24 h of air exposure. The mild performance degradation may be due to the adsorption of oxygen and moisture on the VPTe surface or VPTe/dielectric interface. Better performance is expected for P_80_Te phosphorene‐based heterostructure [41, 42].

Furthermore, a high specific detectivity (D*) of 1.86 × 10^11^ Jones was obtained from VPTeNS‐based phototransistor under 530 nm laser illumination (Equations S2 and S3) [39], highlighting their promising optoelectronic applications (Figure 4h). However, both measured charge mobilities were found to be much lower than their pristine values due to extrinsic factors of FET fabrication and operation, where contact resistance is a crucial limitation. Extremely high contact resistance was introduced between violet phosphorene nanosheets due to point contact [40], misalignment of the material interface, undesirable contact formation, or potential oxidation of the contact point [7, 41, 42, 43, 44]. The measurement of phosphorene‐based devices is extremely difficult due to large contact resistance, causing damage to phosphorene channel materials under ambient conditions.

**FIGURE 5:**
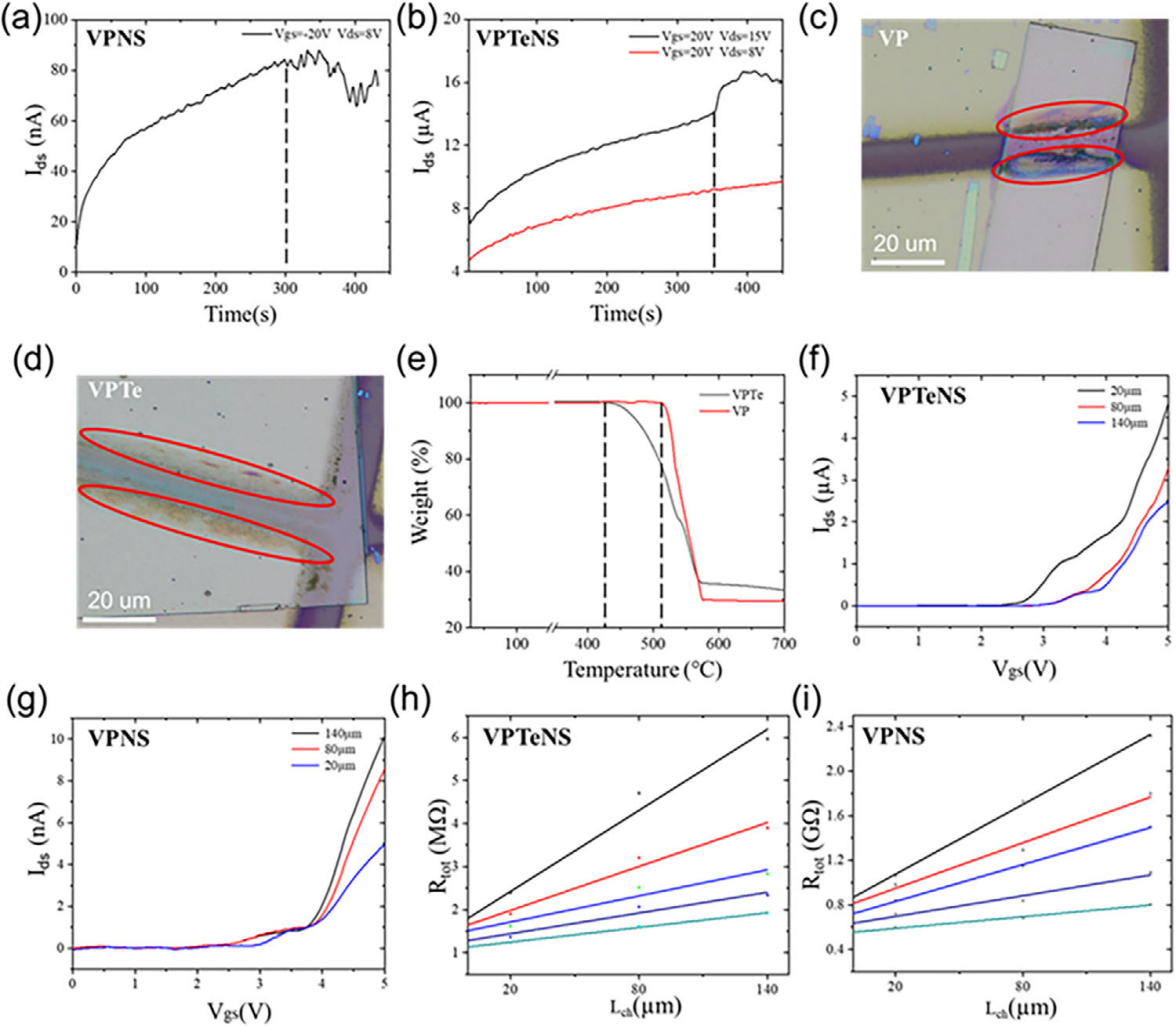
Real‐time current variation of (a) VPNS‐based FET with V_gs_ = −20 V and V_ds_ = 8 V and (b) VPTeNS‐based FET with V_gs_ = 20 V and V_ds_ = 8 V (red) and 15 V (black) under ambient conditions. Optical images of (c) VPNS‐based FETs after measurement according to (a) and (d) VPTeNS‐based FET after measurement according to (b). (e) TGA of VP (red) and VPTe (black) crystals. (f) Output characteristic curves of VPTeNS‐based FET with different channel lengths. (g) Output characteristic curves of VPNS with different channel lengths. Total and contact resistance extracted and fitted using the TLM method of (h) VPTeNS and (i) VPNS channel structures.

The VPNS‐based FET was found to be easily damaged after 300 s of measurements with V_g_ = −20 V and V_ds_ = 8 V (Figure 5a,c). However, the VPTeNS‐based FET was found to be extremely robust; no damage was observed even after measurements for 450 s with V_g_ = 20 V and V_ds_ = 8 V. The VPTeNS‐based FET channel was observed to be damaged only when V_ds_ was increased to 15 V for 350 s measurements (Figure 5b,d). The VPTeNS‐based FET has been found to be much more stable than VPNS counterparts during measurements under ambient conditions, although the starting decomposition temperature of VPTe (422 °C) was found to be lower than that of VP (517 °C) from TGA measurements (N_2_ heating rate of 10 °C min^−^^1^, Figure 5e). The higher performance stability of VPTeNS‐based FET under ambient conditions is due to decreasing contact resistance between channel and electrode materials caused by tellurium substitution, resulting in less generated heat. The contact resistance was roughly deduced from output characteristic curves of VPNS and VPTeNS‐based FETs with different channel lengths (Figure 5f,g) [42]. The contact resistance was extracted from Equation S4. The total and contact resistance of VPNS and VPTeNS were roughly extracted via the TLM method [45, 46, 47, 48, 49], shown in Figure 5h,i, respectively. The contact resistance (R_cont_) of VPTeNS was then extracted to be about 1.13–1.8 MΩ (Figure 5f), three orders of magnitude smaller than that of VPNS (550–860 MΩ). The higher performance stability of VPTeNS‐based FET under ambient conditions has been demonstrated to be attributed to the lower contact resistance of VPTeNS and the electrode due to tellurium substitution [50, 51].

## 3 Conclusion

High‐quality violet tellurium phosphorus crystals have been produced by a modified CVT method, whose crystal structure has been characterized by SC‐XRD with the assistance of Raman, TEM, and elemental analysis to be similar to that of VP, where P9 sites were found to be occupied by mixed Te1/P9 sites. An extra 123 cm^−1^ peak was observed in VPTe crystals, which might be due to localized defect modes caused by tellurium doping or triggering split P‐P vibrational modes. The tellurium substitution has been demonstrated to convert p‐type violet phosphorene into high‐performance n‐type violet tellurium phosphorene. The electron mobility of VPTeNS‐based FET (75.56 nm thickness) was measured to be 134.12 cm^2^ V^−1^ s^−1^, significantly higher than that of the VPNS one (hole mobility: 2.04 cm^2^ V^−1^ s^−1^, thickness: 66.82 nm) under ambient conditions. The VPTeNS‐based FET has been demonstrated to be much more robust than the VPNS‐based one during measurements under ambient conditions, even though the starting decomposition temperature of VPTe (422 °C) was found to be lower than that of VP (517 °C). The VPTeNS‐based FET was found to have much lower contact resistance (1.13–1.80 MΩ) than that of the VPNS‐based one (550–860 MΩ). The tellurium substitution of VP has been demonstrated to significantly lower the contact resistance between VPNS and Ti/Au electrodes, resulting in successful measurements of violet phosphorene‐based FET under ambient conditions.

## 4 Experimental Section/Methods

### 4.1 Synthesis of Violet Tellurium Phosphorus Single Crystals

A mixture of 470 mg of amorphous red phosphorus (Aladdin.99.999%), 200 mg of tin powder (Macklin.99.9%), and 600 mg of tellurium tetraiodide powder (Macklin.99.99%) was sealed in a 75 mm long quartz tube with an inner diameter of 14 mm and a wall thickness of 2 mm with a vacuum of 10–6 mbar. The quartz tube was then placed horizontally in a muffle furnace. The quartz tube was gradually heated to 630 °C in a muffle furnace for 10 h. The sample was kept at this temperature for 5 h, then cooled to 570 °Cat a rate of 10 °C per hour. The tube was cooled from 570 °C to 490 °C for approximately 200 h, and then cooled naturally to room temperature.

### 4.2 Single Crystal X‐Ray Diffraction

Single crystals of violet tellurium phosphorus with regular shape and no obvious cracks were selected, cut to 150 µm to fit the machine holder, and the single crystal X‐ray diffraction data were collected with Mo Kα rays on a Bruker D8 Venture Photon III diffractometer (λ = 0.71073 Å). The structure was solved by direct methods and refined by full matrix least squares against F2. All calculations were carried out by the program package of SHELXTL ver. 6.2 and Olex 2 version 1.2.10. Crystal data of P_80_Te: monoclinic, space group P 2/n (13), a = 9.1903 Å, b = 9.1174 Å, c = 21.8094 Å, β = 97.745^°^, V = 1810.8(2) Å^3^, Z = 1, T = 150 K, 24582 reflections were collected, R1 = 0.0701, wR^2^ = 0.1632. More details are presented in Table S1 and Table S2. CSD‐2449898 contains the supplementary crystallographic data presented in this paper. These data can be obtained free of charge from The Cambridge Crystallographic Data Centre via https://www.ccdc.cam.ac.uk/structures. More details are presented in Table S1 and Table S2.

### 4.3 Preparation of VPNS and VPTeNS‐Based FETs

The samples were mechanically exfoliated with blue tape and transferred onto a silicon substrate with 300 nm SiO_2_ as the dielectric layer. Copper mesh (Gilder Grids) was used as a mask plate for the source and drain. Ti/Au (10 nm/50 nm) was then deposited using e‐Beam evaporation deposition system (HHV, Auto‐500e). The electrical properties of a device were measured under ambient conditions using the Keithley 4200 A Semiconductor Parameter Analyzer.

[Further details of the crystal structure investigation may be obtained from the Fachinformationszentrum Karlsruhe, 76344 Eggenstein‐Leopoldshafen (Germany), on quoting the depository number CSD‐2449898]

## Author Contributions

**Zhuorui Wen**: conceptualization, investigation, writing – original draft, methodology, software, data curation, formal analysis, validation, visualization. **Xuewen Zhao**: conceptualization, methodology, supervision, data curation. **Wenju Zhou**: software, validation. **Wenqi Mu**: investigation. **Rui Zhai**: project administration. **Jiazheng Lin**: data curation. **Xin Liu**: resources. **Kerui Hu**: formal analysis. **Zhiqi Zhang**: formal analysis. **Guodong Meng**: resources. **Yonghong Cheng**: supervision. **Huiyang Gou**: validation. **Jinying Zhang**: conceptualization, writing – review and editing, project administration, resources, funding acquisition, methodology, supervision, formal analysis.

## Funding

National Natural Science Foundation of China (Grant No. 22175136). State Key Laboratory of Electrical Insulation and Power Equipment (Grant No.EIPE23127).

## Conflicts of Interest

The authors declare no conflicts of interest.

## Supporting information




**Supporting File**: advs77560‐sup‐0001‐SuppMat.docx.

## Data Availability

All data generated or analyzed during this study are included in this published article and its supplementary information files.
