# Supplementary material for: High‐Performance Violet Phosphorene‐Based Device via Tellurium Doping‐Induced Contact Resistance Reduction
Source: Adv Sci (Weinh). 2026 Sep 27:e77560. Online ahead of print. doi: 10.1002/advs.77560 (PMC13616332; doi:10.1002/advs.77560)
Supplement: Supplementary file 1 [file ADVS-9999-e77560-s001.docx]

Supporting Information

High-Performance Violet Phosphorene-based device via Tellurium Doping-Induced Contact Resistance Reduction

Zhuorui Wen^#^, Xuewen Zhao^#^, Wenju Zhou, Wenqi Mu, Rui Zhai, Jiazheng Lin, Xin Liu, Kerui Hu, Zhiqi Zhang, Guodong Meng, Yonghong Cheng, Huiyang Gou, Jinying Zhang^*^

**Experimental Session**

**Scanning electron microscopy:** Morphology of violet tellurium phosphorus structures were taken by a FEI Quanta 250F scanning electron microscope.

**Powder X-ray diffraction:** X-ray diffraction patterns were obtained from a Bruker D2 PHASER using Cu/Kαradiation (λ=1.5418 Å) at 40 kV and 30 mA.

**Raman spectroscopy:** Raman spectroscopy was taken in a back-scattering geometry using a single monochromator with a microscope (Reinishaw inVia) equipped with CCD array detector (1024×256 pixels, cooled to -70ºC) and an edge filter. Single pieces of violet tellurium phosphorus or violet phosphorus crystals were adopted for Raman scattering with excitation laser of 633nm.

**TEM, HRTEM and SAED measurements:** TEM, HRTEM images and SAED patterns were acquired using Talos F200X electron microscope with an acceleration voltage of 200 kV. The violet tellurium phosphorus was sonicated in ethanol for 15 min and then dropped onto copper grids coated with ultrathin amorphous carbon films and then dried under ambient conditions.

**X-ray photoelectron spectroscopy:** Bulk VP and VPTe crystals were tested at 400 W using a Thermo Fisher Xi+ and a double anode aluminum/magnesium target. The surface of the samples were cleaned by plasma etching prior before measurements.

**Device parameter deduction**

**Charge mobility:** The charge mobility of the VPTeNS and VPNS based FETs was calculated according to Eq. S1.

$\mu=\frac{dI_{\mathrm{ds}}}{dV_{g}}\frac{L_{\mathrm{ch}}}{W_{\mathrm{ch}}C_{\mathrm{ox}}V_{\mathrm{ds}}}$………………………………………………………………………Eq. S1

The parameter dI_ds_/dV_g_, representing the transconductance, was determined from the slope of the transfer characteristic curve. 𝐿_𝑐h_ is the conducting channel length and 𝑊_𝑐h_ is the channel width. 𝑉_ds_ is the drain-source voltage. The value of the gate capacitance per unit area C_ox_, plays a crucial regulating role in the device characteristics and is calculated as C_ox_= 𝜺_0_𝜺_𝑟_/𝑑, where 𝜺_0_ and 𝜺_𝑟_ are the dielectric constants of the free space and SiO_2_ layers, respectively, and 𝑑 is the thickness of the gate dielectric layer.

**Performance parameters of photodetectors:** The specific detectivity $D^{*}$and responsibility of 2D materials-based photodetectors was calculated according to Eq. S2 and Eq. S3.

$D^{*}=\frac{\sqrt{A_{d}\triangle f}}{NEP}=R\frac{\sqrt{A_{d}\triangle f}}{i_{n}}$………………………………………………………………… Eq. S2

$R=\frac{I_{ph}}{P_{inc}A_{d}/A_{l}}=\frac{I_{ph}}{\bar{P_{inc}}}$………………………………………………………………………Eq. S3

$A_{d}$ is Active Device Area, $i_{n}$ is Noise current, $\triangle f$ is noise bandwidth of the measurement system, R is response rate of the detector, $I_{ph}$ is photo-responsive current, $\bar{P_{inc}}$ is effective incident optical power.

**DFT resistance:** The contact resistance was extracted from Eq. S4.

$R_{tot}=2R_{cont}+R_{unit}*\frac{L_{ch}}{W_{ch}}$………………………………………………………….…Eq. S4

$R_{tot}$ is the total resistance. R_cont_ is the contact resistance between channel material and electrode, R_unit_ is the resistance per unit length of the material, and $L_{ch}$ and $W_{ch}$ are channel length and channel width, respectively.

**Figure S1.** Synthesis parameters for violet tellurium phosphorus crystal growth.

**Figure S2.** As-produced violet tellurium phosphorus products grown with different feedstock ratios.

**Figure S3.** Violet tellurium phosphorus crystals produced with different temperature and cooling rates.

**Figure S4.** Optical image of a violet tellurium phosphorus polycrystal.

**Figure S5.** Optical images of (a)VPNS-based FET and (b) VPTeNS-based FET with different channel lengths.

**Figure S6.** Photocurrent of VPTeNS under different incident light power density.

**Figure S7.** (a) diffuse reflectance spectra, (b) valence band XPS, and (c) deduced band edges of violet tellurium phosphorus and pristine violet phosphorus.

**Figure S8.** Transfer characteristic curves of P_80_Te phosphorene nanosheet-based FET devices (a) before air exposure, (b) after 12 h of exposure to air, and (c) after 24 h of exposure to air. Optical images of the device (d) before air exposure and (e) after 24 h of air exposure.

**Table S1.** Crystal data and structure refinement of P80Te single crystal

| empirical formula | P80 Te |
| --- | --- |
| Formula weight | 2605.20 |
| temperature /K | 150 |
| crystal system | monoclinic |
| space group | P 2/n |
| a /Å | 9.1903(6) |
| b /Å | 9.1174(6) |
| c /Å | 21.8094(14) |
| α/° | 90 |
| β /° | 97.745(2) |
| γ /° | 90 |
| volume /Å3 | 1810.8(2) |
| Z | 1 |
| calculated density /g cm-3 | 2.389 |
| μ /mm‑1 | 2.207 |
| Crystal size/mm3 | 0.25 × 0.2 × 0.05 |
| Radiation | MoKα (λ = 0.71073) |
| F(000) | 1252.0 |
| 2Θ range for data collection /° | 3.77 to 55.006 |
| Index ranges | -11 ≤ h ≤ 11, 0 ≤ k ≤ 9, -28 ≤ l ≤ 28 |
| Reflections collected | 24582 |
| Independent reflections | 4140 [Rint = 0.0562, Rsigma = 0.0392] |
| Data/restraints/parameters | 4140/0/190 |
| Goodness-of-fit on F2 | 1.214 |
| Final R indexes [I>=2σ(I)] | R1 = 0.0701, wR2 = 0.1600 |
| Final R indexes [all data] | R1 = 0.0767, wR2 = 0.1632 |
| Largest diff. peak/hole / e Å-3 | 1.71/-1.07 |

**Table S2.** Atomic coordinates for P_80_Te crystal

| Atom | x | y | z | U(eq) |
| --- | --- | --- | --- | --- |
| Te1 | 2545.4(19) | 8221(2) | 7005.1(8) | 8.9(3) |
| P1 | 8804.2(18) | -2998.0(19) | 6813.6(8) | 8.2(3) |
| P2 | 6919.7(18) | -2230.6(19) | 6165.0(8) | 7.5(3) |
| P3 | 6071.8(19) | -519.5(19) | 6727.1(8) | 8.2(3) |
| P4 | 7490.4(19) | 1447(2) | 6679.8(8) | 9.7(3) |
| P5 | 5724.7(19) | 3242(2) | 6695.7(8) | 9.3(3) |
| P6 | 3785.3(18) | 1788(2) | 6743.1(8) | 8.4(3) |
| P7 | 4200.3(18) | 147.1(19) | 6043.3(8) | 7.7(3) |
| P8 | 5258.1(18) | 1470.3(19) | 5332.8(7) | 6.7(3) |
| P9 | 7623.1(18) | 1363.3(19) | 5663.1(8) | 7.6(3) |
| P10 | 5323.2(17) | 3723.8(19) | 5683.5(8) | 7.2(3) |
| P11 | 2956.9(18) | 4082.9(19) | 5441.7(8) | 7.0(3) |
| P12 | 1917.0(18) | 2800.1(19) | 6162.6(8) | 7.3(3) |
| P13 | 1298.3(18) | 4472.7(19) | 6803.3(8) | 7.6(3) |
| P14 | 3129.0(18) | 6007(2) | 6733.6(8) | 9.0(3) |
| P15 | 2841.2(17) | 6377.7(19) | 5728.6(8) | 6.5(3) |
| P16 | 448.6(17) | 6573.1(19) | 5442.7(7) | 6.4(3) |
| P17 | -576.8(18) | 5317.0(19) | 6168.3(8) | 7.8(3) |
| P18 | 306.2(17) | 8897.2(19) | 5717.7(8) | 6.3(3) |
| P19 | 264.0(19) | 8889(2) | 6722.0(8) | 9.0(3) |
| P20 | -2063.3(18) | 9045.2(19) | 5436.3(8) | 6.9(3) |

**Table S3.** Electrical properties of pristine and doped violet phosphorene-based FET devices.

| channel material | Type | Mobility  (cm^2^ V^−1^ s^−1^) | on/off ratio | subthreshold swing  (mV/dec) | Ref. |
| --- | --- | --- | --- | --- | --- |
| VPTe (P_80_Te) | n | 134.12 | 896 | 109 | This work |
| VPSb | p | 58.96 | ~10^3^ | Not mentioned | ^[3]^ |
| VPAs | n | 137.06 | 664 | 114 | ^[2]^ |
| VP | p | 4.07 | 9.8 | 130 | ^[2]^ |
| VP | p | 2.308 | 150 | Not mentioned | ^[1]^ |

[1]	Y. Wang, M. Jin, M. Gu, X. Zhao, J. Xie, G. Meng, Y. Cheng, J. He, J. Zhang, *Nanoscale* **2023**, 15, 12406.

[2]	R. Zhai, Z. Wen, X. Zhao, J. She, M. Gu, F. Bu, C. Huang, G. Meng, Y. Cheng, J. Zhang, *Nano-Micro Letters* **2026**, 18.

[3]	F. Baumer, Y. Ma, C. Shen, A. Zhang, L. Chen, Y. Liu, D. Pfister, T. Nilges, C. Zhou, *ACS Nano* **2017**, 11, 4105.
